# Treatment-dependent and independent prognostic value of inflammation-based nutritional indices in immune checkpoint inhibitor-based therapy for esophageal cancer

**DOI:** 10.1007/s10388-026-01243-x

**Published:** 2026-08-28

**Authors:** Hiroki Matsuda, Tomoki Makino, Shuichiro Hara, Shigeto Nakai, Takaomi Hagi, Kota Momose, Kotaro Yamashita, Takuro Saito, Koji Tanaka, Atsushi Takeno, Tsuyoshi Takahashi, Masaaki Motoori, Yukinori Kurokawa, Hidetoshi Eguchi, Yuichiro Doki

**Affiliations:** 1 Department of Gastroenterological Surgery, Graduate School of Medicine, The University of Osaka, 2-2-E2, Yamada-Oka, Suita, Osaka, 565-0871 Japan; 2 Department of Surgery, NHO Osaka National Hospital, Osaka, Japan; 3 Department of Surgery, Osaka General Medical Center, Osaka, Japan

**Keywords:** Esophageal squamous cell carcinoma, Immune checkpoint inhibitor, Glasgow prognostic score, Platelet-to-lymphocyte ratio, Real-world evidence

## Abstract

**Background:**

Phase III trials have established the efficacy of immune-based therapies for unresectable advanced or recurrent esophageal squamous cell carcinoma (ESCC). However, real-world evidence comparing regimens and clinically applicable prognostic biomarkers remains limited.

**Methods:**

We retrospectively evaluated 245 patients with unresectable advanced/recurrent ESCC treated with first-line 5-fluorouracil plus cisplatin combined with nivolumab or pembrolizumab (CF + ICI; n = 114), first-line ipilimumab plus nivolumab (Ipi + Nivo; n = 46), or nivolumab monotherapy as second-line or later treatment (Nivo; n = 85). Efficacy, safety, and survival outcomes were assessed, focusing on inflammation-based nutritional indices and multivariate prognostic modelling.

**Results:**

The objective response rates were 56%, 53%, and 27%, and the median overall survival was 26, 73, and 13 months in the CF + ICI, Ipi + Nivo, and Nivo groups, respectively. No treatment-related deaths occurred. In multivariate analyses, Glasgow Prognostic Score (GPS) ≥ 2 independently predicted poor survival across all regimens. High platelet-to-lymphocyte ratio (PLR) was independently associated with poor survival only in chemotherapy-free ICI regimens (Ipi + Nivo and Nivo). A pooled model showed a significant interaction between treatment regimen and PLR (p = 0.047).

**Conclusions:**

In real-world practice, GPS provides regimen-independent prognostic stratification, whereas PLR shows treatment-dependent prognostic relevance, supporting its potential utility for biomarker-guided and individualized treatment selection in ESCC.

**Supplementary Information:**

The online version contains supplementary material available at https://doi.org/10.1007/s10388-026-01243-x.

## Introduction

Esophageal cancer is the seventh leading cause of cancer-related mortality worldwide [1]. Esophageal squamous cell carcinoma (ESCC) is the predominant histological subtype, accounting for approximately 90% of cases [2]. The prognosis of patients with unresectable or metastatic ESCC remains poor, with a median overall survival (OS) of 8–10 months [3–7]. The programmed cell death protein 1 (PD-1) pathway plays a critical role in regulating host immune defense against tumors and represents a key mechanism of tumor immune evasion. Inhibition of the PD-1 pathway enhances antitumor T-cell activity by blocking interactions between the PD-1 receptor and its ligands [8, 9].

Recent phase III trials have established the efficacy of PD-1 immune checkpoint inhibitors (ICIs) in unresectable advanced or recurrent esophageal cancer. The ATTRACTION-3 trial demonstrated the superiority of nivolumab over taxane-based chemotherapy in second-line therapy [10]. In first-line treatment, 5-fluorouracil plus cisplatin (CF) plus pembrolizumab improved survival in the KEYNOTE-590 trial [11], whereas CF plus nivolumab and nivolumab plus ipilimumab regimens were superior to CF alone in the CheckMate-648 trial [12]. Furthermore, the RATIONALE-306 trial demonstrated that CF plus tislelizumab provided a survival benefit over CF plus placebo [13]. Based on these pivotal studies, current standard first-line treatment in Japan consists of CF-based chemotherapy combined with anti-PD-1 antibodies or dual immune checkpoint inhibition, and nivolumab monotherapy is approved as second-line or later therapy for patients not previously treated with ICIs. However, programmed death-ligand 1 (PD-L1) is the only biomarker currently used in clinical practice and is insufficient for optimal treatment selection. Therefore, additional biomarkers are needed to stratify patients and distinguish therapeutic efficacy between chemotherapy plus ICIs and dual-ICI regimens [14, 15].

Inflammation-based nutritional indices are potential biomarkers for immunotherapy across various malignancies [14], but most studies have indicated that these indices primarily function as general prognostic factors, and few have examined whether their prognostic significance differs by treatment regimen [16–18]. In this study, we evaluated the clinical outcomes of ICI-based therapies, specifically CF combined with pembrolizumab or nivolumab, the combination of ipilimumab and nivolumab, and nivolumab monotherapy, in patients with unresectable advanced or recurrent esophageal cancer. We also investigated whether inflammation-based nutritional indices could serve as prognostic biomarkers and whether their impact varies according to treatment regimen.

## Materials and methods

### Study design and patients

This single-center retrospective observational study included patients with unresectable advanced or recurrent ESCC. At our institution, 114 patients received CF combined with nivolumab or pembrolizumab (CF + ICI group) and 46 patients received ipilimumab plus nivolumab (Ipi + Nivo group) as first-line treatment between December 2017 and August 2025. Treatment selection was based on both tumour- and patient-related factors. In general, CF + ICI was preferred for patients with bulky or rapidly progressive tumours, symptomatic disease (e.g., dysphagia or pain), locally advanced tumours (cT3br–T4), or liver metastases, provided they had a good performance status and were considered fit for cisplatin-based chemotherapy. In contrast, ipilimumab plus nivolumab was preferentially selected for patients with a relatively low tumour burden, PD-L1-positive tumours, advanced age, cisplatin intolerance (e.g., creatinine clearance < 40 mL/min), or those who preferred outpatient treatment. An additional 85 patients received nivolumab monotherapy as second-line or later treatment between February 2014 and November 2021. Clinical data were retrospectively collected from electronic medical records at our institution. Patients were censored at the date of last follow-up.

### Definitions of sarcopenia and inflammation-based nutritional indices

The psoas muscle index (PMI) was defined as the total cross-sectional area of the bilateral psoas muscles at the third lumbar vertebral level divided by the square of the patient’s height. Sarcopenia was defined as PMI < 6.36 cm^2^/m^2^ in men and PMI < 3.92 cm^2^/m^2^ in women, based on previously reported CT-based criteria for Asian adults [4, 14]. Inflammatory and nutritional markers were assessed using laboratory data obtained from peripheral blood samples collected immediately before treatment initiation. The platelet-to-lymphocyte ratio (PLR) was calculated as the absolute platelet count divided by the absolute lymphocyte count. The neutrophil-to-lymphocyte ratio was calculated as the absolute neutrophil count divided by the absolute lymphocyte count. The lymphocyte-to-monocyte ratio was calculated as the absolute lymphocyte count divided by the absolute monocyte count. The prognostic nutritional index was calculated using the following formula: 10 × serum albumin level + 0.005 × total lymphocyte count. The C-reactive protein (CRP)-to-albumin ratio was calculated as the serum CRP level divided by the serum albumin level. The systemic immune-inflammation index was calculated as: (platelet count × neutrophil count)/(lymphocyte count × 1000). When analyzed as categorical variables, the median value of the nutritional indicator within each treatment group was used as the cutoff.

The Glasgow Prognostic Score (GPS) was calculated based on serum CRP and albumin levels. Patients with both elevated CRP (≥ 1.0 mg/dL) and hypoalbuminemia (albumin < 3.5 g/dL) were assigned a score of 2, those with one of these abnormal values were assigned a score of 1, and those with neither abnormality were assigned a score of 0.

### Treatment regimens

Treatment with CF plus pembrolizumab consisted of 80 mg/m^2^ cisplatin on day 1, 800 mg/m^2^ fluorouracil on days 1–5, and 200 mg pembrolizumab on day 1 every 3 weeks[11] Treatment with CF plus nivolumab consisted of 80 mg/m^2^ cisplatin on day 1, 800 mg/m^2^ fluorouracil on days 1–5, and nivolumab at 240 mg on days 1 and 15 or 480 mg on day 1 every 4 weeks [12]. Treatment with Ipi + Nivo consisted of 1 mg/kg ipilimumab on day 1 and nivolumab at 360 mg on days 1 and 22 or 240 mg on days 1, 15, 29 every 6 weeks. [12] Nivolumab monotherapy consisted of nivolumab at 480 mg on day 1 or 240 mg on days 1, 15 every 4 weeks [10]. Dose modifications and treatment discontinuation were at the discretion of the treating physician. Treatment was continued until disease progression, unacceptable toxicity, or patient refusal.

### Evaluation of treatment response and adverse events

Although a follow-up schedule was not specified in this study, tumor response was assessed every 4–8 weeks by computed tomography [3, 19], endoscopy, and PET-CT[19–21] (as needed) according to Response Evaluation Criteria in Solid Tumors (RECIST) version 1.1 and categorized as a complete response (CR), partial response (PR), stable disease (SD), or progressive disease (PD) [20, 21]. In patients without measurable lesions, tumor response was categorized as CR, non-CR/non-PD, or PD. Non-evaluable patients were regarded as non-responders. The objective response rate (ORR) was assessed only in patients with measurable lesions and was defined as the proportion of patients whose best overall treatment response was CR or PR. The disease control rate (DCR) was defined as the proportion of patients who achieved CR, PR, SD, or non-CR/non-PD. Progression-free survival (PFS) was defined as the time from treatment initiation to disease progression or death from any cause and was censored at the date of surgery in patients who underwent conversion surgery. Overall survival (OS) was measured from treatment initiation to death from any cause. Adverse events were graded according to the Common Terminology Criteria for Adverse Events (CTCAE) version 5.0.

### Statistical analysis

Continuous variables are presented as median (range), and categorical variables are presented as number (percentage). Survival curves were obtained using the Kaplan–Meier method and compared with the log-rank test. Univariable and multivariable analyses were performed using Cox proportional hazards models. For each treatment group, two separate multivariable models were specified a priori: one evaluating GPS and the other evaluating PLR. Each model was adjusted for age, sex, ECOG performance status, and the number of metastatic organs, irrespective of their statistical significance in the univariable analyses. Separate models were constructed to avoid multicollinearity among inflammation-based nutritional indices sharing component variables and to reduce the risk of overfitting. For group-specific analyses, continuous variables were dichotomized into high and low categories using the median value within each treatment group. Subsequently, all patients were pooled, and a multivariate Cox proportional hazards model was constructed including treatment group (categorical variable), PLR (continuous variable), and an interaction term between treatment group and PLR to evaluate whether the prognostic effect of the PLR differed across treatment groups. Age (continuous variable), Sex (categorical variable), ECOG PS (continuous variable), and number of metastatic organs (continuous variable) were included as clinically relevant covariates. The significance of the interaction term was assessed using the likelihood ratio test. These analyses were conducted in an exploratory manner. All statistical tests were two-sided, and *p* < 0.05 was considered significant. Statistical analyses were performed using JMP version 17.0 (SAS Institute, Cary, NC, USA). For comparisons among the three GPS categories, an overall log-rank test was performed, followed by pairwise log-rank tests with Holm adjustment for multiple comparisons.

## Results

### Patient characteristics

A total of 245 patients were included in this study: 114 in the CF + ICI group, 46 in the Ipi + Nivo group, and 85 treated with nivolumab monotherapy. The CF + ICI group consisted of 73 patients treated with CF plus pembrolizumab and 41 patients treated with CF plus nivolumab. No participants were excluded. Patient characteristics are summarized in Table 1. The median age in the CF + ICI group was 70 years (range 31–85 years) and most patients were male (77%). The majority of patients in this group (94%) had a good ECOG PS (i.e., 0–1). Unresectable advanced disease was present in 75 patients (66%), whereas 39 patients (34%) had recurrent disease. Most patients (54%) in this group had one metastatic organ and 43% had two or more metastatic organs; only 3% had a primary or locally recurrent lesion without distant metastasis. In the Ipi + Nivo group, the median age was 74 years (range 52–87 years) and most patients were male (91%). A good ECOG PS was observed in 94% of patients. Unresectable advanced disease was present in 17 patients (37%), whereas 29 patients (63%) had recurrent disease. Most patients (54%) in this group had one metastatic organ and 44% had two or more metastatic organs; only 2% had a primary or locally recurrent lesion without distant metastasis. In the Nivo group, the median age was 67 years (range 49–89 years) and most patients were male (79%). All patients had a good ECOG PS. Unresectable advanced disease was present in 24 patients (28%), whereas 60 patients (71%) had recurrent disease. Less than half (47%) of patients in this group had one metastatic organ and 51% had two or more metastatic organs; only 2% had a primary or locally recurrent lesion without distant metastasis. CF + ICI and Ipi + Nivo were administered as first-line treatments in all patients, whereas nivolumab monotherapy was administered as second-line or later treatment in all but one patient. Inflammatory and nutritional indices by treatment regimen are shown in Table 2.

**Table 1:** Patient characteristics

Characteristics	CF + ICI (N = 114)	Ipi + Nivo (N = 46)	Nivolumab (N = 85)
Age, years	70 (31–85)	74 (52–87)	67 (49–89)
*Sex*			
Male	88 (77)	42 (91)	67 (79)
Female	26 (23)	4 (9)	18 (21)
*ECOG performance status score*			
0	87 (76)	33 (72)	68 (80)
1	20 (18)	10 (22)	17 (20)
≥ 2	7 (6)	3 (6)	0
*Tumor status*			
Stage IV	75 (66)	17 (37)	24 (28)
Recurrence after surgery	39 (34)	29 (63)	60 (71)
Other	0 (0)	0 (0)	1 (1)
*Number of metastatic organs*			
0	3 (3)	1 (2)	2 (2)
1	62 (54)	25 (54)	40 (47)
≥ 2	49 (43)	20 (44)	43 (51)
*Metastatic site*			
Lymph nodes	105 (92)	35 (76)	68 (80)
Lung	31 (27)	17 (37)	36 (42)
Liver	21 (18)	5 (11)	19 (22)
Pleura/peritoneum	9 (8)	4 (9)	3 (4)
Local/primary	72 (63)	20 (43)	16 (19)
*Treatment line*			
1st	114 (100)	46 (100)	1 (1)
2nd	-	-	59 (69)
3rd or later	-	-	25 (29)
*Tumor proportion score*			
> 0%, ≤ 1%	25 (22)	7 (15)	-
> 1%, ≤ 10%	18 (16)	10 (22)	-
> 10%	22 (19)	11 (24)	-
N.A.^a^	49 (43)	18 (39)	85 (100)
*Sarcopenia*			
Yes	89 (78)	33 (72)	58 (68)
No	25 (22)	13 (28)	27 (32)
*Smoking status*			
Never	36 (32)	9 (20)	61 (72)
Current or former	75 (66)	37 (80)	24 (28)
Unknown	3 (2)	0 (0)	0 (0)
*Drinking status*			
Never or occasional	38 (33)	11 (24)	36 (42)
Current or former	71 (62)	35 (76)	49 (58)
Unknown	5 (5)	0 (0)	0 (0)

Values are given as n (%) or median (range)

^a^N.A., data not available

**Table 2:** Inflammatory and nutritional indices by treatment regimen

	CF + ICI (N = 114)	Ipi + Nivo (N = 46)	Nivolumab (N = 85)
PNI^a^	41.0 (23.1–57.8)	44.0 (29.1–54.2)	43.5 (28.4–54.2)
NLR^b^	4.10 (0.78–25.64)	3.03 (0.84–11.70)	3.81 (1.41–19.93)
LMR^c^	2.64 (0.71–6.38)	3.35 (0.90–7.51)	2.69 (0.48–7.33)
PLR^d^	228.9 (68.8–1398.6)	191.0 (75.5–637.6)	211.2 (80.2–969.0)
CAR^e^	0.09 (0.01–4.04)	0.20 (0.01–8.62)	0.10 (0.01–4.55)
GPS^f^, 0/1/2/N.A.^g^	60/34/19/1	25/18/3	48/26/11
SII^h^	986.7 (132.9–10,000.0)	684.5 (162.4–4255.7)	811.4 (274.1–5696.7)

^a^*PNI* prognostic nutritional index

^b^*NLR* neutrophil-to-lymphocyte ratio

^c^*LMR* lymphocyte-to-monocyte ratio

^d^*PLR* platelet-to-lymphocyte ratio

^e^*CAR* C-reactive protein to albumin ratio

^f^*GPS* Glasgow prognostic score

^g^*SII* systemic immune-inflammation index

^h^N.A., data not available

### Adverse events

Adverse events are summarized in Online Resource 1. In the CF + ICI group, adverse events of any grade and adverse events of grade 3 or greater occurred in 98 patients (86%) and 66 patients (58%), respectively. The most common adverse events of any grade were neutropenia (n = 53, 46%), decreased appetite (n = 39, 34%), and hyponatremia (n = 37, 32%). The most frequent adverse events of grade 3 or greater were neutropenia (n = 43, 38%), hyponatremia (n = 12, 11%), and anemia (n = 12, 11%). In the Ipi + Nivo group, adverse events of any grade and adverse events of grade 3 or greater occurred in 32 patients (70%) and 17 patients (37%), respectively. The most common adverse events of any grade were rash (n = 22, 47%), hypothyroidism (n = 13, 28%), and adrenal insufficiency (n = 11, 24%). Adverse events of grade 3 or greater mainly included adrenal insufficiency (n = 8, 17%) and interstitial lung disease (n = 4, 9%). In the nivolumab monotherapy group, adverse events of any grade and adverse events of grade 3 or greater occurred in 24 patients (28%) and 7 patients (8%), respectively. The most common adverse events of any grade were hypothyroidism (n = 14, 16%), diarrhea (n = 12, 14%), and rash (n = 8, 9%). Adverse events of grade 3 or greater included interstitial lung disease (n = 4, 5%) and hyponatremia (n = 3, 4%).

### Treatment efficacy

The best overall responses are summarized in Table 3. In the CF + ICI group, CR and PR were observed in 11 patients (10%) and 39 patients (34%), respectively, whereas PD occurred in 17 patients (15%). The ORR was 56%, and the DCR was 83%. In the Ipi + Nivo group, CR and PR were observed in 6 patients (13%) and 14 patients (30%), respectively, whereas PD occurred in 14 patients (30%). The ORR and DCR were 53% and 70%, respectively. In the nivolumab monotherapy group, CR and PR were observed in 9 patients (11%) and 14 patients (16%), respectively, whereas PD occurred in 37 patients (44%). The ORR was 27%, and the DCR was 56%.

**Table 3:** Antitumor activity according to treatment regimen

Outcome	CF+ICI (N=114)	Ipi+Nivo (N=46)	Nivolumab (N=85)
Complete response	11 (10)	6 (13)	9 (11)
Partial response	39 (34)	14 (30)	14 (16)
Stable disease	20 (18)	4 (9)	20 (24)
Progressive disease	17 (15)	14 (30)	37 (44)
Non-CR/non-PD	25 (22)	8 (17)	5 (6)
Not evaluable	2 (2)	0 (0)	0 (0)
Objective response rate	45 (56)	20 (53)	19 (27)
Disease control rate	95 (83)	32 (70)	48 (56)

Values are given as n (%)

*Objective response rate was assessed only in patients with measurable lesions

### Survival analysis

**Fig. 1:**
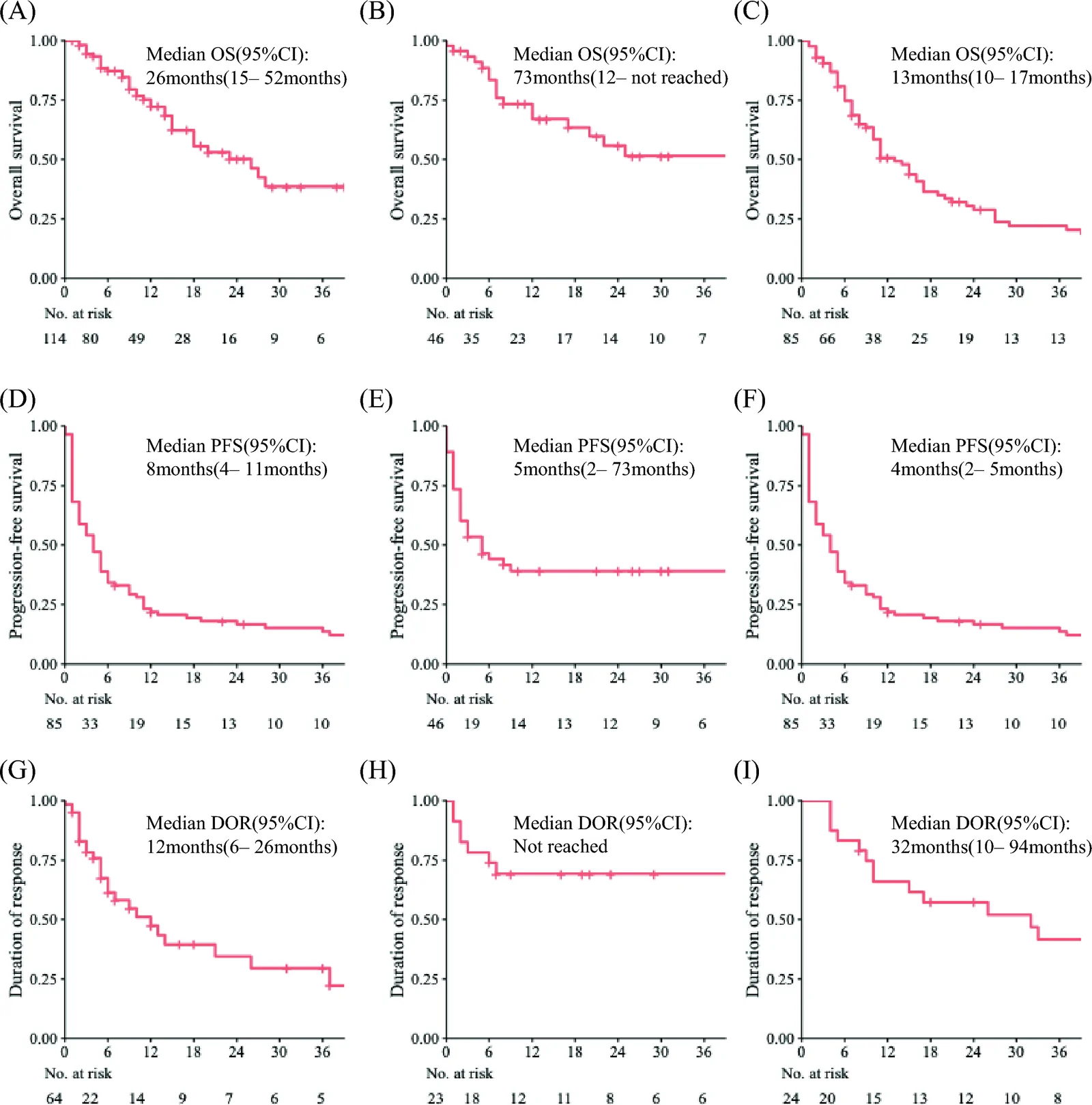
Treatment efficacy and survival outcomes according to treatment regimen. (**A**– **C)** Kaplan–Meier curves for overall survival in patients treated with (**A)** CF + ICI, (**B)** Ipi + Nivo, and (**C)** Nivo alone. (**D**–**F**) Kaplan–Meier curves for progression-free survival in patients treated with (**D)** CF + ICI, (**E)** Ipi + Nivo, and (**F)** Nivo alone. (**G**–**I)** Kaplan–Meier curves for duration of response among responders in patients treated with (**G)** CF + ICI, (**H)** Ipi + Nivo, and (**I)** Nivo alone

Survival outcomes for each treatment group are shown in Fig. 1. In the CF + ICI group, the median follow-up period among censored patients was 9 months. The median OS was 26 months (95% confidence interval [CI], 15–52 months), the median PFS was 8 months (95% CI, 4–11 months), and the median duration of response (DOR) was 12 months (95% CI, 6–26 months). In the Ipi + Nivo group, the median follow-up period among censored patients was 15.5 months. The median OS was 73 months (95% CI, 12 months to not reached), the median PFS was 5 months (95% CI, 2–73 months), and the median DOR was not reached. In the nivolumab monotherapy group, the median follow-up period among censored patients was 21 months. The median OS was 13 months (95% CI, 10–17 months), the median PFS was 4 months (95% CI, 2–5 months), and the median DOR was 32 months (95% CI, 10–94 months).

**Fig. 2:**
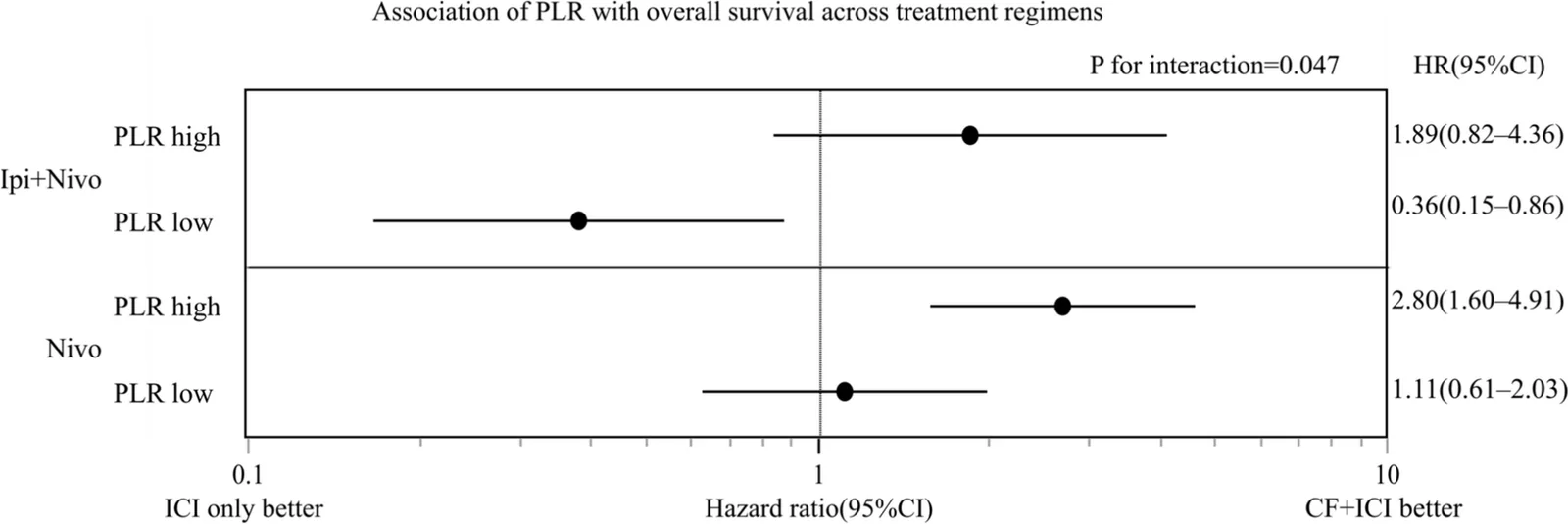
Interaction between pretreatment PLR and treatment regimen for overall survival. Patients were pooled across the three treatment regimens and divided into PLR-low and PLR-high groups using the overall median PLR. In each PLR group, adjusted HRs (95% CIs) for OS were estimated using multivariable Cox models including treatment regimen, age, sex, performance status, and number of metastatic organs, and displayed as a forest plot. The interaction between PLR and treatment regimen was tested in a multivariable Cox model including the interaction term (P for interaction = 0.047, likelihood ratio test)

The results of the univariable Cox proportional hazards analyses for OS are summarized in Online Resource 2. In multivariable models adjusted for age, sex, ECOG performance status, and the number of metastatic organs, GPS ≥ 2 was independently associated with poorer OS across all treatment groups (CF + ICI: hazard ratio [HR], 3.06; 95% confidence interval [CI], 1.34–7.01; p = 0.008; Ipi + Nivo: HR, 4.30; 95% CI, 1.05–17.5; p = 0.042; Nivo: HR, 3.73; 95% CI, 1.43–9.71; p = 0.007; Table 4A). In separate multivariable models adjusted for the same covariates, a high PLR was independently associated with poorer OS in the Ipi + Nivo and nivolumab monotherapy groups (Ipi + Nivo: HR, 4.31; 95% CI, 1.29–14.4; p = 0.018; Nivo: HR, 2.63; 95% CI, 1.53–4.54; p < 0.001; Table 4B), but not in the CF + ICI group (HR, 1.28; 95% CI, 0.66–2.52; p = 0.462; Table 4B). In the pooled analysis, the interaction between treatment regimen and PLR remained statistically significant (likelihood ratio test, p = 0.047; Fig. 2).

**Table 4:** Multivariate Cox proportional hazards analyses of overall survival according to inflammation-based nutritional indices

		CF + ICI		Ipi + Nivo		Nivolumab	
		Multivariate		Multivariate		Multivariate	
		HR (95% CI)	*p*-value	HR (95% CI)	*p*-value	HR (95% CI)	*p*-value
*(A) Model evaluating GPS*							
Age	≥ Median	1.57(0.81–3.06)	0.181	0.79(0.27–2.30)	0.662	0.78(0.45–1.36)	0.389
Sex	Male	1.55(0.67–3.57)	0.302	0.79(0.16–3.78)	0.764	0.95(0.47–1.94)	0.888
ECOG Performance	≥ 1	1.95(0.83–4.62)	0.127	1.49(0.44–5.11)	0.524	1.78(0.81–3.93)	0.152
Status							
Number of	≥ 2	1.25(0.65–2.40)	0.501	0.83(0.33–2.14)	0.705	1.31(0.76–2.27)	0.332
metastatic organs							
GPS^a^	≥ 2	3.06(1.34–7.01)	0.008	4.3(1.05–17.5)	0.042	3.73(1.43–9.71)	0.007
*(B) Model evaluating PLR*							
Age	≥ Median	1.45(0.74–2.81)	0.277	0.7(0.24–1.99)	0.502	0.74(0.44–1.25)	0.261
Sex	Male	1.22(0.55–2.71)	0.618	0.45(0.08–2.62)	0.371	1.91(0.95–3.86)	0.07
ECOG Performance	≥ 1	2.34(1.01–5.40)	0.047	0.77(0.23–2.63)	0.681	2.31(1.15–4.62)	0.019
Status							
Number of	≥ 2	1.48(0.78–2.82)	0.234	0.96(0.36–2.55)	0.936	1.28(0.78–2.12)	0.33
metastatic organs							
PLR^b^	≥ Median	1.28(0.66–2.52)	0.462	4.31(1.29–14.4)	0.018	2.63(1.53–4.54)	< 0.001

^a^GPS, Glasgow Prognostic Score; Hazard ratios (HRs) indicate the effect of the index within each treatment group

^b^PLR, platelet-to-lymphocyte ratio; Hazard ratios (HRs) indicate the effect of the index within each treatment group

**Fig. 3:**
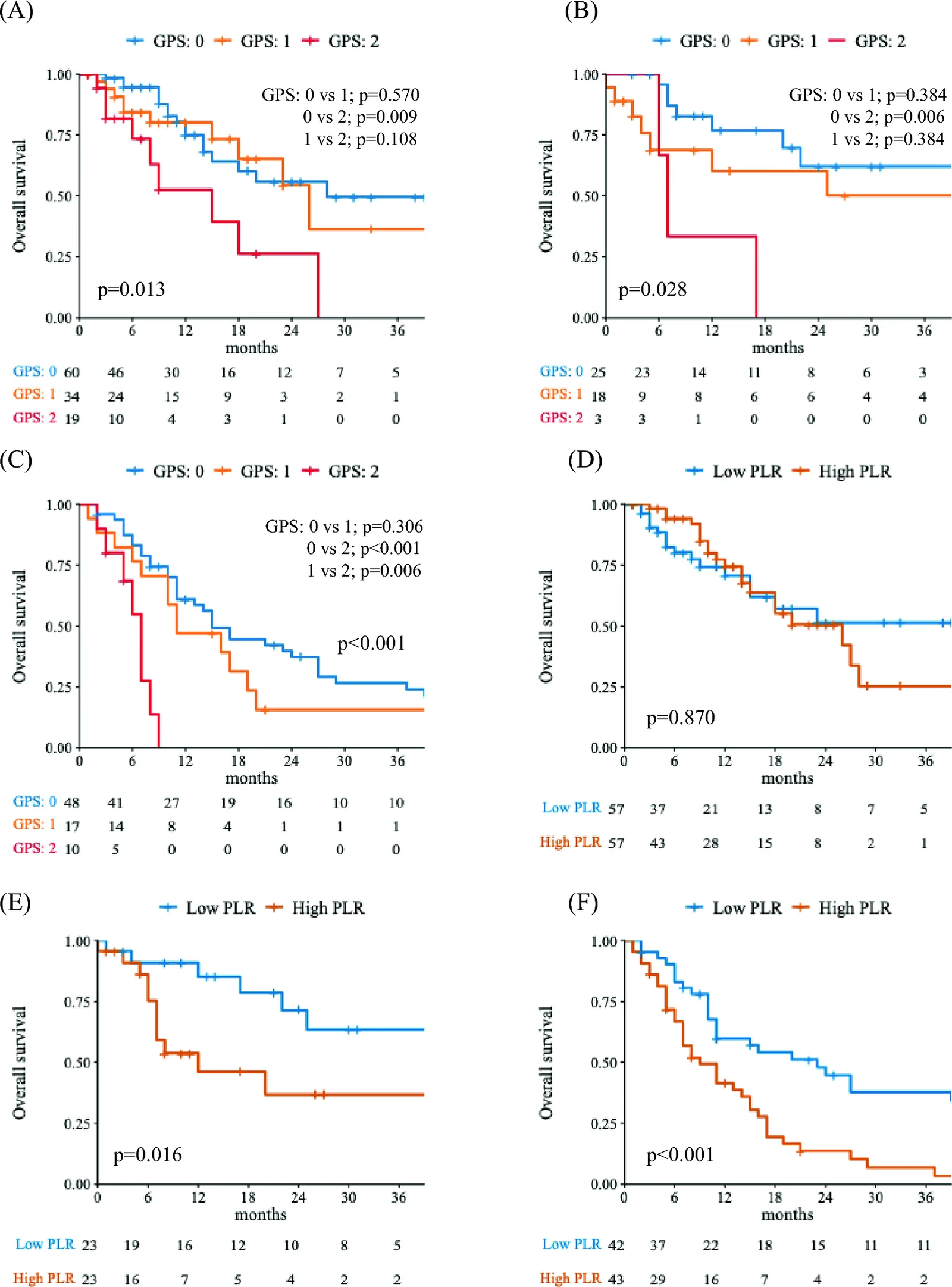
Prognostic impact of the Glasgow Prognostic Score (GPS) and platelet-to-lymphocyte ratio (PLR) according to treatment regimen. (**A**–**C)** Kaplan–Meier curves for Overall survival according to GPS in patients treated with (**A)** CF + ICI, (**B)** Ipi + Nivo, and (**C)** Nivo alone. An overall log-rank test across the three GPS categories was performed, followed by pairwise log-rank tests with Holm adjustment for multiple comparisons. (**D**–**F)** Kaplan–Meier curves for Overall survival according to PLR in patients treated with (**D)** CF + ICI, (**E)** Ipi + Nivo, and (**F)** Nivo alone

Survival curves stratified by GPS and PLR are shown in Fig. 3 and Online Resource 3. The median OS was significantly shorter in patients with GPS 2 than in those with GPS 0 across all treatment groups (CF + ICI: GPS 2 vs 1 vs 0, 9 vs. 26 vs. 28 months; Ipi + Nivo: 7 vs. 73 months vs. not reached; nivolumab: 6 vs. 11 vs. 15 months) (Fig. 3A-C). Similarly, the median PFS was shorter in patients with GPS 2 than in those with GPS 0 across all treatment groups (CF + ICI: GPS 2 vs 1 vs 0, 4 vs. 5 vs. 8 months; Ipi + Nivo: 1 vs. 3 vs. 9 months; nivolumab: 2 vs. 5 vs. 4 months) (Online Resource 3A-C). When stratified by PLR, the median OS was shorter in patients with a high PLR than in patients with a low PLR after ICI-based regimens without chemotherapy (Ipi + Nivo: 12 months vs. not reached, *p* = 0.016; nivolumab: 9 vs. 23 months, *p* < 0.001) (Fig. 3D-F). The median PFS was significantly shorter in patients with a high PLR than in patients with a low PLR after these regimens (Ipi + Nivo: 2 months vs. not reached, *p* = 0.008; nivolumab: 2 vs. 6 months, *p* = 0.002) (Online Resource 3D-F). Prognostic analyses according to the occurrence of immune-related adverse events (irAEs) and PD-L1 expression, assessed by the tumor proportion score (TPS), are shown in Online Resource 4 and Online Resource 5. In the CF + ICI group, the occurrence of irAEs (grade1 or higher) was not associated with survival outcomes. In contrast, the occurrence of irAEs (grade1 or higher) was significantly associated with improved OS and PFS in both the Ipi + Nivo group and nivolumab monotherapy group. Among patients with available TPS data, TPS ≥ 1% was associated with longer OS and PFS only in the Ipi + Nivo group.

## Discussion

In this single-center retrospective study, we evaluated the clinical outcomes of three ICI-based regimens for unresectable advanced or recurrent ESCC. CF + ICI and Ipi + Nivo as first-line treatment demonstrated comparable response rates with distinct safety profiles, whereas nivolumab monotherapy showed modest efficacy in second-line (or later) settings. Inflammation-based nutritional indices, particularly the GPS, consistently stratified prognosis regardless of treatment regimen. In contrast, the prognostic impact of the PLR differed by treatment modality and was significant only in ICI-based regimens without chemotherapy. To the best of our knowledge, this is the first study to demonstrate the treatment-dependent prognostic relevance of the PLR in patients with ESCC receiving ICI-based therapy, highlighting the potential clinical utility of simple blood-based biomarkers in treatment selection.

The median OS of patients treated with CF + ICI in our cohort was longer than the OS reported in the global KEYNOTE-590 and CheckMate-648 trials, as well as in their Japanese subgroup analyses.[11, 12, 24, 25] Similarly, patients in this study treated with Ipi + Nivo had longer OS and PFS than patients in the CheckMate-648 trial. The observed difference between median OS and median PFS in this group may be due to the short follow-up period, with most patients being censored at an early time point. The incidence of adverse events of grade 3 or greater was lower or comparable to the incidence reported in phase III trials. These favorable outcomes may be explained, at least in part, by differences in patient characteristics, including a higher proportion of patients with good ECOG PS and recurrent disease, as well as treatment selection and subsequent therapies in routine clinical practice. In addition, the relatively short follow-up period may have resulted in immature survival estimates, particularly in the dual ICI (Ipi + Nivo) group.

The GPS has been widely reported to be a robust prognostic indicator across various malignancies, including those treated with ICIs. [26, 27] In the present study, the GPS remained a significant prognostic factor across all treatment groups, suggesting that it primarily reflects a treatment-independent impact related to systemic inflammation and nutritional status rather than serving as a predictive biomarker for specific regimens. On the other hand, the PLR reflects the balance between tumor-promoting inflammatory activity and the antitumor immune response. Although previous studies demonstrated an association between a high PLR and poor prognosis in patients receiving ICIs,[28–30] its treatment-specific relevance was unclear. Our findings demonstrated that the PLR has treatment-dependent prognostic significance and is associated with outcomes after ICI monotherapy and dual-ICI therapy, but not after ICI combined with chemotherapy. These results suggest that the PLR may complement the GPS by providing additional information relevant to treatment selection. Thus, combined assessment of the GPS and PLR may offer a practical framework for treatment stratification in ESCC. The GPS may estimate overall prognosis regardless of treatment modality, whereas the PLR may help guide the selection of chemotherapy plus ICI or dual ICI-based regimens.

This study has several limitations. The retrospective, single-center design may have introduced selection bias and residual confounding, and the follow-up period was limited in some patients. The median overall survival observed in the ipilimumab plus nivolumab group should be interpreted with caution because of the relatively short follow-up period and the high proportion of censored observations. The differences in baseline characteristics among treatment groups may have affected GPS and PLR values and, consequently, their prognostic impact, and data on PD-L1 expression were not available for a substantial proportion of cases. Nevertheless, this study represents a relatively large real-world cohort and provides a rare comparative evaluation of prognostic factors across multiple ICI-based regimens. Prospective, multi-institutional studies with longer follow-up are warranted to validate these findings and clarify the role of the GPS and PLR in guiding treatment selection.

## Conclusion

In patients with unresectable advanced or recurrent ESCC, ICI-based regimens demonstrated distinct efficacy and safety profiles depending on treatment modality. Inflammation-based nutritional indices, particularly the GPS, consistently stratified prognosis across treatment regimens, whereas the prognostic value of the PLR differed according to the presence or absence of chemotherapy. These findings suggest that simple, routinely available biomarkers may aid in optimizing treatment selection between chemotherapy plus ICI and dual-ICI strategies and highlight the need for prospective, multi-institutional studies to validate the clinical utility of the GPS and PLR as prognostic biomarkers.

## Supplementary Information

Below is the link to the electronic supplementary material.

Supplementary file 1 (PDF 66 KB)

Supplementary file 2 (PDF 136 KB)

Supplementary file 3 (DOCX 273 KB)

## Data Availability

The datasets used in this study are not publicly available because they contain patient information, but they are available from the corresponding author on reasonable request.
