# Supplementary material for: Treatment-dependent and independent prognostic value of inflammation-based nutritional indices in immune checkpoint inhibitor-based therapy for esophageal cancer
Source: Esophagus. 2026 Aug 28;23(4):869–78. doi: 10.1007/s10388-026-01243-x (PMC13723529; doi:10.1007/s10388-026-01243-x)
Supplement: Supplementary file 1 [file 10388_2026_1243_MOESM1_ESM.pdf]

Online Resource 1. Adverse events according to treatment regimen

Adverse events	CF+ICI (N=114)		Ipi+Nivo (N=46)		Nivolumab (N=85)	
	Any grade	≥Grade 3	Any grade	≥Grade 3	Any grade	≥Grade 3
All events	98 (86)	66 (58)	32 (70)	17 (37)	49 (58)	18 (21)
Neutropenia	53 (46)	43 (38)	0 (0)	0 (0)	0 (0)	0 (0)
Leukopenia	50 (44)	27 (24)	1 (2)	0 (0)	0 (0)	0 (0)
Loss of appetite	39 (34)	11 (10)	3 (6)	2 (4)	3 (4)	1 (1)
Hyponatremia	37 (32)	12 (11)	5 (11)	2 (4)	3 (4)	3 (4)
Renal dysfunction	32 (28)	5 (4)	5 (11)	2 (4)	2 (2)	0 (0)
Fatigue	32 (28)	1 (1)	4 (8)	1 (2)	5 (6)	0 (0)
Anemia	31 (27)	12 (11)	2 (4)	1 (2)	6 (7)	0 (0)
Nausea	27 (24)	2 (2)	1 (2)	0 (0)	2 (2)	0 (0)
Stomatitis	20 (18)	4 (4)	1 (2)	0 (0)	1 (1)	1 (1)
Thrombocytopenia	20 (18)	4 (4)	0 (0)	0 (0)	0 (0)	0 (0)
Diarrhea	16 (14)	5 (4)	6 (13)	1 (2)	12 (14)	0 (0)
Hypoalbuminemia	14 (12)	0 (0)	3 (6)	0 (0)	3 (4)	0 (0)
Lymphopenia	13 (11)	6 (5)	0 (0)	0 (0)	0 (0)	0 (0)
Rash	10 (9)	0 (0)	22 (47)	2 (4)	8 (9)	0 (0)
Abnormal hepatic function	7 (6)	3 (3)	5 (11)	2 (4)	3 (4)	1 (1)
Adrenal insufficiency	4 (4)	1 (1)	11 (24)	8 (17)	1 (1)	0 (0)
Interstitial lung disease	3 (3)	2 (2)	7 (15)	4 (9)	7 (8)	4 (5)
Neuropathy	3 (3)	0 (0)	1 (2)	0 (0)	0 (0)	0 (0)
Hypothyroidism	2 (2)	0 (0)	13 (28)	2 (4)	14 (16)	0 (0)
Glucose intolerance	1 (1)	0 (0)	2 (4)	1 (2)	2 (2)	0 (0)
Hyperthyroidism	0 (0)	0 (0)	2 (4)	1 (2)	1 (1)	0 (0)
Arthritis	0 (0)	0 (0)	2 (4)	0 (0)	0 (0)	0 (0)
Hypopituitarism	0 (0)	0 (0)	1 (2)	0 (0)	0 (0)	0 (0)
