# Supplementary material for: Treatment-dependent and independent prognostic value of inflammation-based nutritional indices in immune checkpoint inhibitor-based therapy for esophageal cancer
Source: Esophagus. 2026 Aug 28;23(4):869–78. doi: 10.1007/s10388-026-01243-x (PMC13723529; doi:10.1007/s10388-026-01243-x)
Supplement: Supplementary file 2 [file 10388_2026_1243_MOESM2_ESM.pdf]

Online Resource 2. Univariate Cox proportional hazards analyses of overall survival

		**CF+ICI**		**Ipi+Nivo**		**Nivolumab**	
		Univariate		Univariate		Univariate	
		HR (95% CI)	*p*-value	HR (95% CI)	*p*-value	HR (95% CI)	*p*-value
Age	≥Median	1.23 (0.65-2.30)	0.528	0.70 (0.27-1.79)	0.458	0.93 (0.56-1.52)	0.763
Sex	Male	1.37 (0.62-2.98)	0.435	0.90 (0.20-3.96)	0.888	1.30 (0.68-2.50)	0.424
ECOG Performance Status	≥1	2.20 (0.98-4.95)	0.056	1.12 (0.36-3.44)	0.849	2.15 (1.11-4.15)	0.021
Number of metastatic organs	≥2	1.48 (0.79-2.78)	0.226	0.84 (0.33-2.14)	0.715	1.11 (0.68-1.82)	0.675
PNI[a]	≥Median	0.74 (0.39-1.40)	0.354	0.46 (0.18-1.18)	0.106	0.46 (0.28-0.77)	0.003
NLR[b]	≥Median	1.89 (1.00-3.57)	0.049	1.52 (0.59-3.90)	0.382	2.01 (1.22-3.31)	0.006

LMR[c]	≥Median	0.61 (0.32-1.15)	0.124	0.45 (0.17-1.18)	0.105	0.72 (0.44-1.18)	0.191
PLR[d]	≥Median	1.05 (0.56-1.99)	0.872	3.15 (1.16-8.50)	0.024	2.41 (1.43-4.04)	<0.001
CAR[e]	≥Median	1.92 (1.00-3.70)	0.051	1.95 (0.76-5.06)	0.17	1.71 (1.00-2.93)	0.050
GPS[f]	≥2	3.10 (1.49-6.47)	0.006	4.08 (1.13-14.7)	0.032	4.81 (2.04-11.35)	0.001
SII[g]	≥Median	1.55 (0.82-2.92)	0.179	1.22 (0.48-3.10)	0.679	1.80 (1.09-3.00)	0.021

[a]PNI, prognostic nutritional index; [b]NLR, neutrophil-to-lymphocyte ratio; [c]LMR, lymphocyte-to-monocyte ratio; [d]PLR, platelet-to-lymphocyte ratio; [e]CAR, C-reactive protein to albumin ratio; [f]GPS, Glasgow Prognostic Score; [g]SII, systemic immune-inflammation index; CI, confidence interval. Hazard ratios (HRs) indicate the effect of the index within each treatment group.
