# Supplementary material for: Treatment-dependent and independent prognostic value of inflammation-based nutritional indices in immune checkpoint inhibitor-based therapy for esophageal cancer
Source: Esophagus. 2026 Aug 28;23(4):869–78. doi: 10.1007/s10388-026-01243-x (PMC13723529; doi:10.1007/s10388-026-01243-x)
Supplement: Supplementary file 3 [file 10388_2026_1243_MOESM3_ESM.docx]

**Online Resource 3 Prognostic impact of the Glasgow Prognostic Score (GPS) and platelet-to-lymphocyte ratio (PLR) according to treatment regimen**

(A–C) Kaplan–Meier curves for Progression-free survival according to GPS in patients treated with (A) CF+ICI, (B) Ipi+Nivo, and (C) Nivo alone. An overall log-rank test across the three GPS categories was performed, followed by pairwise log-rank tests with Holm adjustment for multiple comparisons. (D–F) Kaplan–Meier curves for progression-free survival according to PLR in patients treated with (D) CF+ICI, (E) Ipi+Nivo, and (F) Nivo alone.

**Online Resource 4 Association between immune-related adverse events and survival outcomes according to treatment regimen.**

IrAE-positive was defined as the occurrence of grade 1 or higher immune-related adverse events (irAEs). Kaplan–Meier curves for overall survival (OS) according to irAE occurrence in patients treated with (A) CF+ICI, (B) Ipi+Nivo, and (C) nivolumab alone, and for progression-free survival (PFS) according to irAE occurrence in patients treated with (D) CF+ICI, (E) Ipi+Nivo, and (F) nivolumab alone.

**Online Resource 5 Association between PD-L1 tumor proportion score and survival outcomes according to treatment regimen.**

Kaplan–Meier curves for overall survival (OS) according to PD-L1 tumor proportion score (TPS) in patients treated with (A) CF+ICI and (B) Ipi+Nivo, and for progression-free survival (PFS) according to TPS in patients treated with (C) CF+ICI and (D) Ipi+Nivo.
